# New Model for Stacking Monomers in Filamentous Actin from Skeletal Muscles of *Oryctolagus cuniculus*

**DOI:** 10.3390/ijms21218319

**Published:** 2020-11-06

**Authors:** Anna V. Glyakina, Alexey K. Surin, Sergei Yu. Grishin, Olga M. Selivanova, Mariya Yu. Suvorina, Liya G. Bobyleva, Ivan M. Vikhlyantsev, Oxana V. Galzitskaya

**Affiliations:** 1 Institute of Protein Research, Russian Academy of Sciences, 142290 Pushchino, Moscow Region, Russia; quark777a@gmail.com (A.V.G.); alan@vega.protres.ru (A.K.S.); syugrishin@gmail.com (S.Y.G.); seliv@vega.protres.ru (O.M.S.); marrruko@yandex.ru (M.Y.S.); 2 Institute of Mathematical Problems of Biology, Russian Academy of Sciences, Keldysh Institute of Applied Mathematics, Russian Academy of Sciences, 142290 Pushchino, Moscow Region, Russia; 3 The Branch of the Institute of Bioorganic Chemistry, Russian Academy of Sciences, 142290 Pushchino, Moscow Region, Russia; 4 State Research Center for Applied Microbiology and Biotechnology, 142279 Obolensk, Moscow Region, Russia; 5 Institute of Theoretical and Experimental Biophysics, Russian Academy of Sciences, 142290 Pushchino, Moscow Region, Russia; liamar@rambler.ru (L.G.B.); ivanvikhlyantsev@gmail.com (I.M.V.)

**Keywords:** actin, monomer, filament, proteolysis, accessible surface area, mass spectrometry, electron microscopy

## Abstract

To date, some scientific evidence (limited proteolysis, mass spectrometry analysis, electron microscopy (EM)) has accumulated, which indicates that the generally accepted model of double-stranded of filamentous actin (F-actin) organization in eukaryotic cells is not the only one. This entails an ambiguous understanding of many of the key cellular processes in which F-actin is involved. For a detailed understanding of the mechanism of F-actin assembly and actin interaction with its partners, it is necessary to take into account the polymorphism of the structural organization of F-actin at the molecular level. Using electron microscopy, limited proteolysis, mass spectrometry, X-ray diffraction, and structural modeling we demonstrated that F-actin presented in the EM images has no double-stranded organization, the regions of protease resistance are accessible for action of proteases in F-actin models. Based on all data, a new spatial model of filamentous actin is proposed, and the F-actin polymorphism is discussed.

## 1. Introduction

At present, much attention is being paid to the study of the mechanisms of aggregation of proteins and peptides, especially the elucidation of the organization of their ordered aggregates [1,2,3,4]. The study of the structure of protein homopolymers is associated with the problem of finding effective inhibitors of fibrillation in amyloid diseases [5,6,7]. Despite intensive research on this issue, there is currently no consistent model describing the molecular mechanism of amyloid fibril formation. Researchers face complex challenges; however, it is possible that the key factor preventing the construction of a unified model of fibril organization is the phenomenon of fibril polymorphism for the same protein or peptide [8,9,10,11].

Here we focus on actin, for which the filamentous form (F-actin) is its functional form [12,13,14]. F-actin is formed as a result of aggregation of the globular form of the actin monomer (G-actin), and this aggregation, in contrast to the formation of amyloids, is reversible [15,16,17]. The ability of actin to polymerize and depolymerize is of great importance for many biological functions, such as muscle contraction, cell migration, organization of the cytoskeleton, and transport of organelles [18,19,20,21,22,23]. The interaction of filamentous actin with myosin is the basis of muscle contraction. The protein titin plays a regulatory role in muscle contraction [24,25,26,27]. It was shown that an increase in Ca^2+^ concentration increases the strength and stability of the N2A region of interactions between titin and filamentous actin [28].

The length of actin filaments varies significantly depending on the type of tissue and localization in cells. For example, the length in sarcomeres is 1.10 ± 0.03 μm, and the length in spectrin network in erythrocyte membrane is 33 ± 5 nm [29,30]. There are about 160 different actin-binding proteins, the function of which is to block, stabilize, cross-link, and disrupt filaments [31,32]. Actin filaments are polar; their ends (slow-growing and fast-growing) differ in the structure and dynamics of polymerization/depolymerization. For the fast-growing end, the capping proteins are gelsolin, CapZ, and adducin [31,33,34], and for the slow-growing end, tropomodulin [35], acumentin [36], and Arp2/3 complex [37]. Tropomodulin has been found in various tissues and cells, and its role is especially important when actin filaments must maintain a constant length [38].

Since the 1950s, intensive studies have been carried out on the structure of monomeric and filamentous actin. Based on the data of X-ray structural analysis of polymer actin, it was suggested that F-actin can be helical [39]. On the basis of EM analysis and data from paper [39], it was first stated that F-actin is a double helix [40]. Since then, the idea of a double-stranded organization of filamentous actin has been generally accepted. The atomic structure of F-actin in the form of a double helix was proposed by Holmes et al. in 1990 [41] based on the fitting of the crystal structure of monomeric G-actin in the X-ray diffraction data obtained for oriented F-actin gels [41]. In this case, it was assumed that the structures of F-actin and actin in combination with DNase I are the same. Two actin molecules interact with each other at the following amino acid residues: 322–325 with 243–245, 286–289 with 202–204, 166–169 and 375 with 41–45. It has also been shown that modification of the amino acid residue H40 prevents the formation of filamentous actin. In the subsequent years, this model was refined and verified [18,42,43]. In the work of Kudryashov et al. [42] the structure of filamentous actin was proposed on the basis of dimeric crystal structures, which were obtained by cross-linking of amino acid residues Q41 and C374 included in two adjacent actin molecules. This model differs from the Holmes ‘s model [41] by the smaller twist of two consecutive actin molecules relative to each other. Consequently, there is a decrease in the distance (from 20 to 3 Å on average) between the following amino acid residues: E205 (OE2) and K291 (NZ), S199 (O) and K291 (NZ), T203 (OG1) and D288 (OD2), G197 (O) and T324 (CG2), S199 (CB) and T324 (OG1), T202 (CG2) and I287 (O), D244 (CB) and K326 (NZ). The structure proposed by Oda et al. [18] suggests that the conformational transition of actin from a monomeric to a filamentous form occurs by a simple rotation of two adjacent actin molecules relative to each other by 20°, which makes actin fibril flat. Oztug Durer et al. [43] showed that the cross-linking of amino acid residues 45 and 169, 47 and 169, and 50 and 169 leads to the destruction of F-actin. The structures of filamentous actin in the double-stranded form listed above may not be entirely correct. It was also shown that archaeal filamentous actin has a single-stranded helical structure [44,45], but bacterial filamentous actin [46], like eukaryotic, has a double-stranded helical structure. It was found that archaeal actin (crenactin) is more similar in amino acid sequence to eukaryotic than to bacterial actin. Usually, in all these models of filamentous actin (eukaryotic, bacterial and archaeal) monomers do not overlap with each other.

The strong polymorphism of F-actin [47,48,49,50,51] justifies the continuation of studies of the morphology of filaments in coordination with alternative interpretations of the structural features of F-actin. It was shown in [51] that actin structures can be divided into four large groups (F-, C-, O-, and G-forms) based on the orientation of two main domains: the outer domain and the inner domain. The outer domain consists of subdomains 1 and 2, and the inner domain consist of subdomains 3 and 4. The F-form was observed in the structures of naked actin and actin filaments in complex with tropomyosin. The C-form was observed in actin filaments decorated with cofilin. The O-form was observed in phosphate-treated crystals of the profilin-actin complexes. The G-form has been observed in the crystal structures of monomeric actin. The F-form and C-form cannot transform to the G-form by thermal fluctuations. So, extensive literature data show that F-actin cannot be described by a single structural model, since this model cannot explain the introduction of cross-links (S-S bonds) inside the filament. These results indicate a high degree of plasticity and heterogeneity of F-actin.

Our latest preliminary experimental data for limited proteolysis, mass spectrometry analysis, X-ray diffraction, and electron microscopy images are inconsistent with the double helical organization of filamentous actin. Thus, it becomes necessary to revise the existing data set in a new way and propose a new model that would satisfy the current experimental data.

## 2. Results and Discussion

### 2.1. Structural Analysis of F-Actin

We were interested in what kind of stacking of actin monomers exist in filamentous structure. For this, we selected those structures in which actin filaments consist of more than eight monomers. This selection includes 20 of these filamentous structures with different number of monomers resolved by electron microscopy (EM) or cryo-EM at different levels of resolutions in different years. For example, 5 out of 20 such structures are given in Table 1.

The root mean square deviations (RMSDs) between the monomers of the five filamentous actin structures were calculated. The values obtained for these structures do not exceed 1.3 Å (Table 1). Noteworthy is the surprisingly low RMSD for the monomers in each filament structure.

### 2.2. Electron Microscopy Analysis

**Figure 1:**
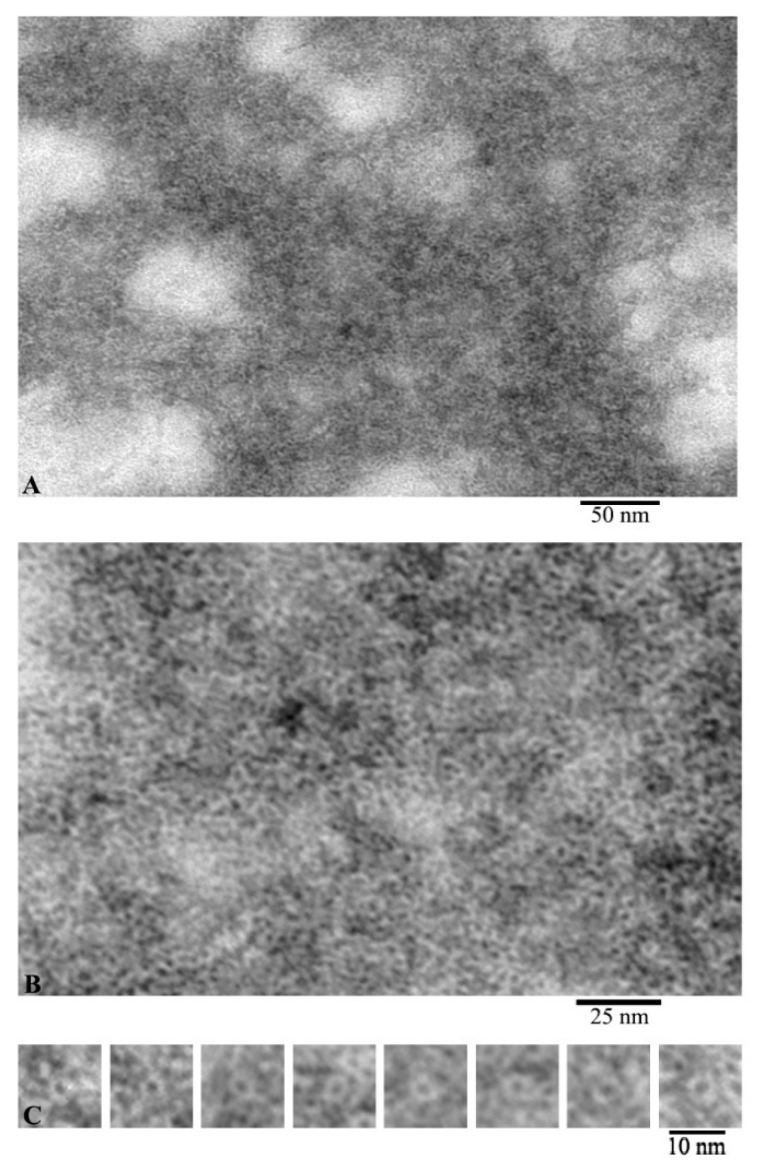
Images of G-actin obtained by electron microscopy. Field (**A**), fragment of high magnification field (**B**) and single molecules of monomeric actin (**C**).

According to the EM analysis data, G-actin forms aggregates of different sizes under conditions of 20 mM TrisHCl, pH 7.8, 5 mM ATP, 0.5 mM β-mercaptoethanol (βME) (Figure 1A). At high magnification, it can be seen that these aggregates are composed of annular particles with a diameter of about 6 nm (Figure 1B). According to the data of X-ray structural analysis, it was found that the monomeric actin molecule has a size of ~5.5 × 5.5 × 3.5 nm. Structurally, G-actin is subdivided into two domains, each of which consists of two subdomains. The domains in the actin molecule are oriented relative to each other in such a way that an open space remains between the subdomains. Since, with negative contrast, the contrast fills all cavities of the object under study, such an object looks like a ring structure. The individual particles with a ring structure are shown in Figure 1C. Since the outer diameter of such particles corresponds to the parameters of an individual actin molecule, it can be argued that we observe individual actin monomers from the front side.

**Figure 2:**
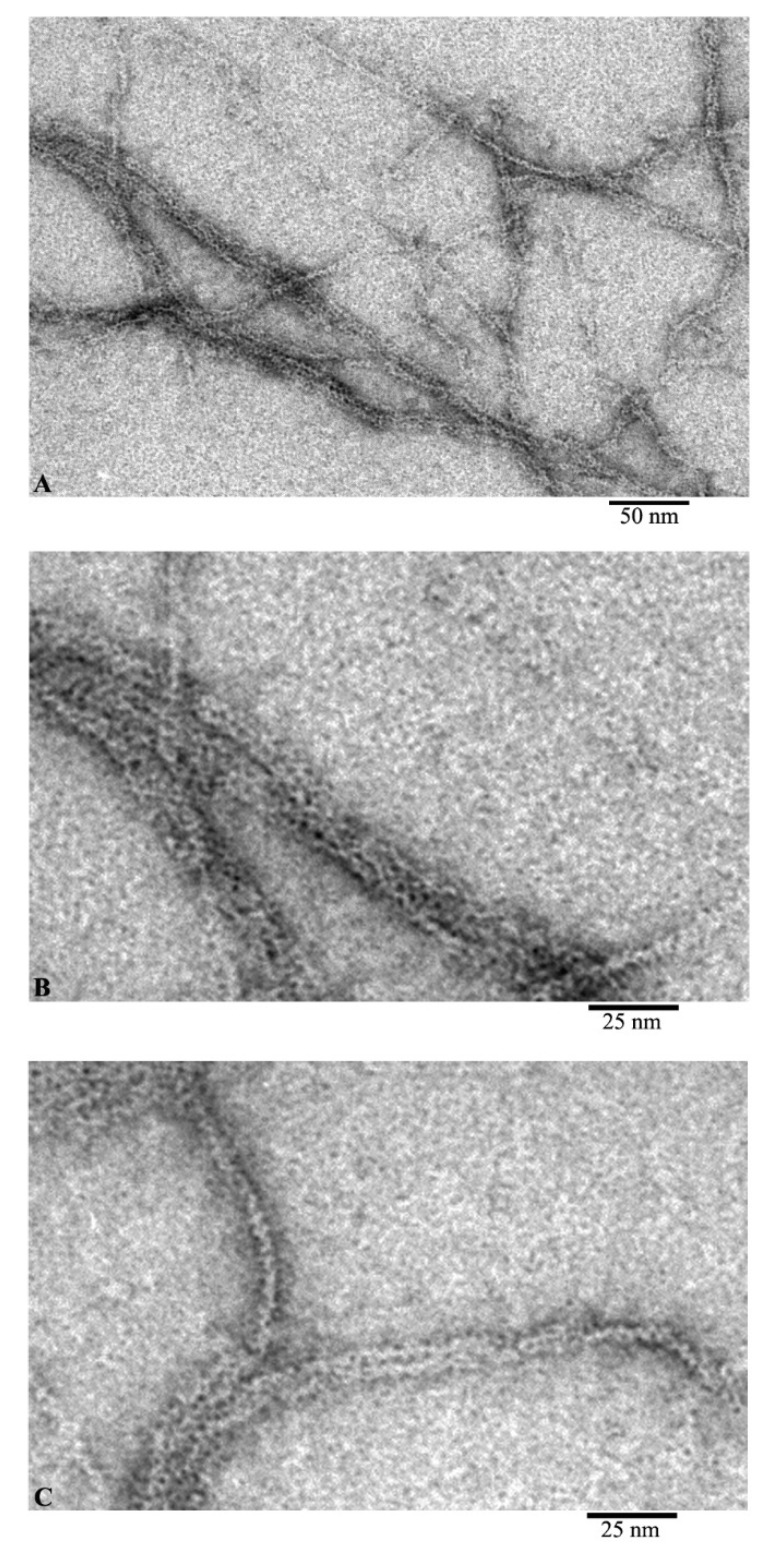
Images of F-actin obtained by electron microscopy. Field (**A**) and fragments of fields of actin filaments: fragments of filaments during adsorption onto the film by the front sides (**B**) and by the side surfaces of actin molecules (**C**).

Using EM analysis of F-actin under the conditions of 20 mM TrisHCl, pH 7.8, 5 mM ATP, 0.5 mM βME, 0.1 M KCl, we observe fibrils 10 and more microns in length (Figure 2A). At higher magnification, it is noticeable that the diameter of the fibrils is either 6–7 nm, or at the inflection points of the fibrils about 3–4 nm (Figure 2B,C). At the same time, with a diameter of 6–7 nm, it is noticeable that the fibril consists of ring-like molecules with a diameter of about 6 nm. No annular particles are observed in the places of inflection; we observe a fibril lying with its lateral surface on the film. In this case, the fibril diameter is about 3–4 nm, which may indicate that F-actin is built from a single chain of monomeric actin molecules.

During the adsorption of G-actin on the film in a lateral orientation, monomeric actin molecules interact with each other with their lateral surfaces in such a way that they slightly overlap each other and morphologically resemble a ladder structure with a fibril diameter of about 3–4 nm, corresponding to the height of a monomeric molecule actin.

**Figure 3:**
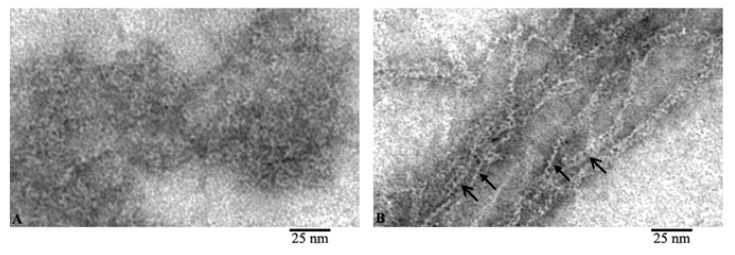
Comparison of G- and F-actin (**A** and **B**, respectively). Filaments during adsorption on the film by the front (open arrow) and lateral (closed arrow) surfaces of actin molecules.

Comparison of the images of G- and F-actin clearly demonstrates that F-actin is formed by the interaction of monomeric actin molecules (Figure 3). It is clearly seen that if the fibril lies on the film with the front side of the molecules, then a fibril with a diameter of about 6 nm is observed (Figure 3B, open arrows). In the places where F-actin bends, actin monomers are adsorbed on the film by the lateral surfaces and structures in the form of a “ladder” are observed (Figure 3B, closed arrows). The diameter in such areas of the fibril is about 3–4 nm. In both projections, the monomers interact by ring to ring slightly overlapping with each other. It is logical to assume that if F-actin consisted of two filaments, then we would observe fibrils only with a diameter of about 6 nm. Thus, the EM analysis data indicate that F-actin is most likely to have a single-stranded organization.

From these images, we can conclude that F-actin is composed of monomers arranged in a ladder pattern (Figure 1, Figure 2 and Figure 3). These images show that the actin filaments are heterogeneous in diameter and are about 7–8 nm in the wide part and about 3–4 nm in the narrow part, which, taking into account the EM resolution, approximately corresponds to the size of the actin monomer. The obtained results do not agree with the generally accepted concepts of the double-stranded organization of F-actin.

### 2.3. Limited Proteolysis and Mass Spectrometry Analysis

Knowledge of the proteolysis-resistant F-actin regions to (the so-called core) helps us to understand the morphology of monomers stacking in the filament.

**Figure 4:**
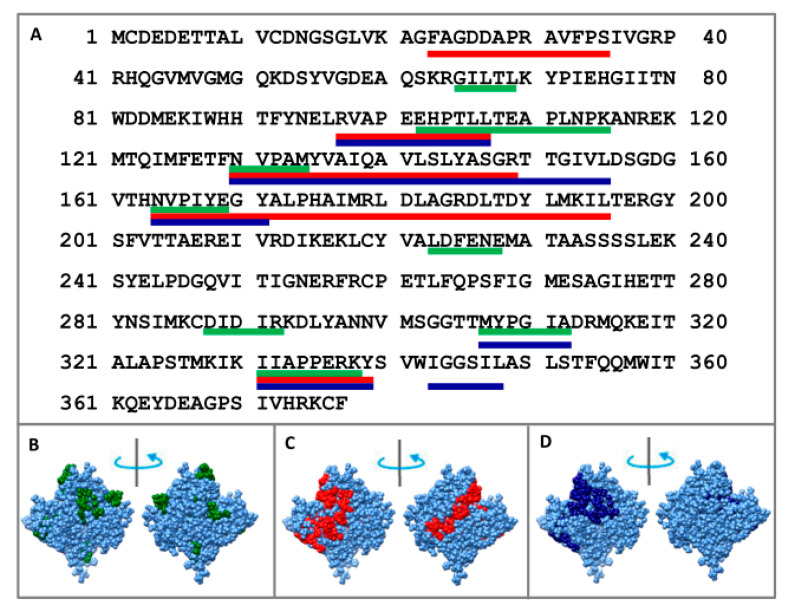
Mass spectrometry analysis of the monomer actin regions protected to the proteolysis treated with mixture of proteases (green lines: 65–69, 102–115, 130–134, 164–167, 223–228, 288–292, 308–312, and 331–338); mass spectrometry analysis of F-actin regions protected to the proteolysis treated with proteinase K (red lines: 23–35, 97–107, 130–149, 164–195, and 331–339) and treated with the mixture of proteases: trypsin, chymotrypsin and proteinase K (blue lines: 97–107, 130–155, 164–171, 307–312, 331–339, and 343–348) indicated on the primary (**A**) and spatial (**B**–**D**) structures of actin.

To determine the core, F-actin was treated in one case with proteinase K, and in the other with a mixture of proteases (trypsin, chymotrypsin and proteinase K). Using mass spectrometry analysis, we determined that proteolysis of F-actin leads to the accumulation of the following peptides: 23–35, 97–107, 130–149, 164–195, and 331–339 (when treated with proteinase K) and 97–107, 130–155, 164–171, 307–312, 331–339, and 343–348 (when treated with the mixture of proteases) (see Figure 4 and Figure S1). One can see that the same peptides (97–107, 331–339) appear in both cases: upon treatment with proteinase K and a mixture of proteases. Monomeric actin regions (65–67, 102–115, 130–134, 164–167, 223–228, 288–292) protected from proteolysis correspond to the terminal parts of the peptides accumulated for filamentous actin. Two regions (308–312 and 331–338) react almost identically to proteolysis in G- and F-actin.

It should be noted that regions 130–149 and 164–195 are amyloidogenic as predicted by the FoldAmyloid program [52,53].

**Figure 5:**
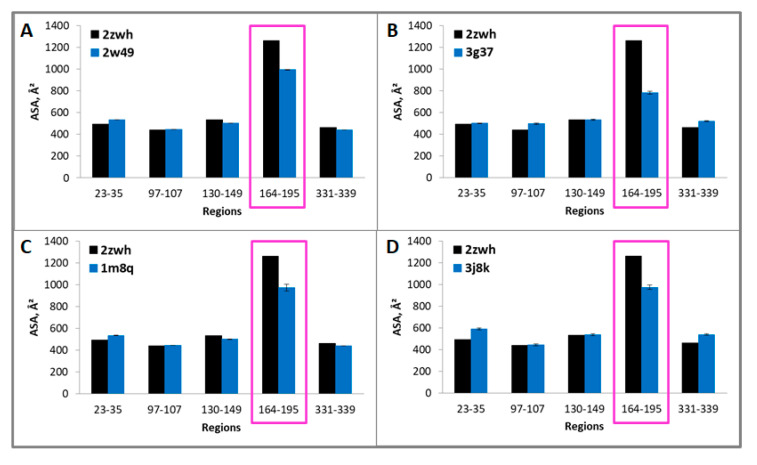
Accessible surface areas of regions 23–35, 97–107, 130–149, 164–195, and 331–339 for monomeric (2zwh) and filamentous (2w49—16 monomers (**A**), 3g37—12 monomers (**B**), 1m8q—14 monomers (**C**), 3j8k—10 monomers (**D**)) actin structures. These regions detected by using mass spectrometry after proteolysis of proteinase K. In the filamentous structures, only region 164–195 (red box) are less accessible to the solvent in comparison with the monomeric actin structure.

**Figure 6:**
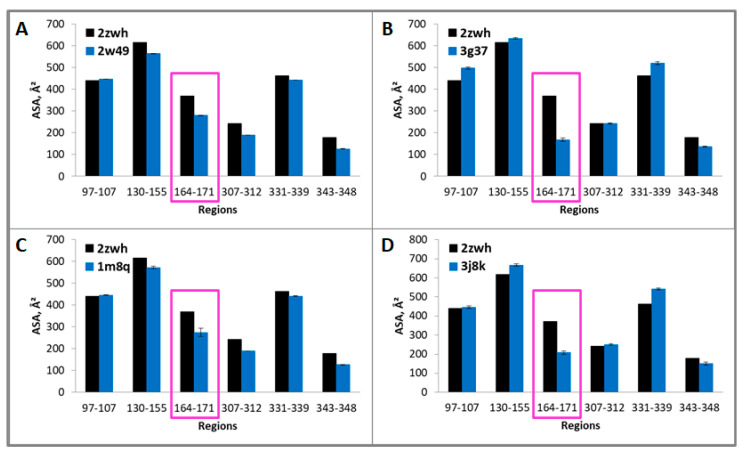
Accessible surface areas of regions 97–107, 130–155, 164–171, 307–312, 331–339, and 343–348 for monomeric (2zwh) and filamentous (2w49—16 monomers (**A**), 3g37—12 monomers (**B**), 1m8q—14 monomers (**C**), 3j8k—10 monomers (**D**)) actin structures. These regions were detected by using mass spectrometry after proteolysis of mixture of proteases: trypsin, chymotrypsin and proteinase K. In the filamentous structures, only region 164–171 (red box) are less accessible to the solvent in comparison with the monomeric actin structure.

Because these regions are resistant to proteolysis, they are part of the core that is inaccessible to the solvent. Therefore, if we calculate the accessible surface areas (ASAs) for G- and F-actin, we should find the difference in these values that is characteristic of a particular actin model. The ASAs of the regions resistant to proteolysis 23–35, 97–107, 130–149, 164–195, 331–339 (proteinase K) and 97–107, 130–155, 164–171, 307–312, 331–339, 343–348 (mixture of proteases) for filamentous (2w49, 1m8q, 3g37, 3j8k, 6bnp) and monomeric (2zwh) actin structure were calculated. In the filamentous structures, only regions 164–195 (proteinase K) and 164–171 (mixture of proteases) are less accessible to the solvent in comparison with the monomeric actin structure (see Figure 5 and Figure 6). A decrease in the accessibility of this area may be due to the double-stranded structure of filamentous actin. No differences in the ASAs for other regions between filamentous and monomeric actin structures were revealed. Thus, the existing filamentous structures of actin do not satisfy the experimental data on the treatment of F-actin with proteinase K and a mixture of proteases.

**Figure 7:**
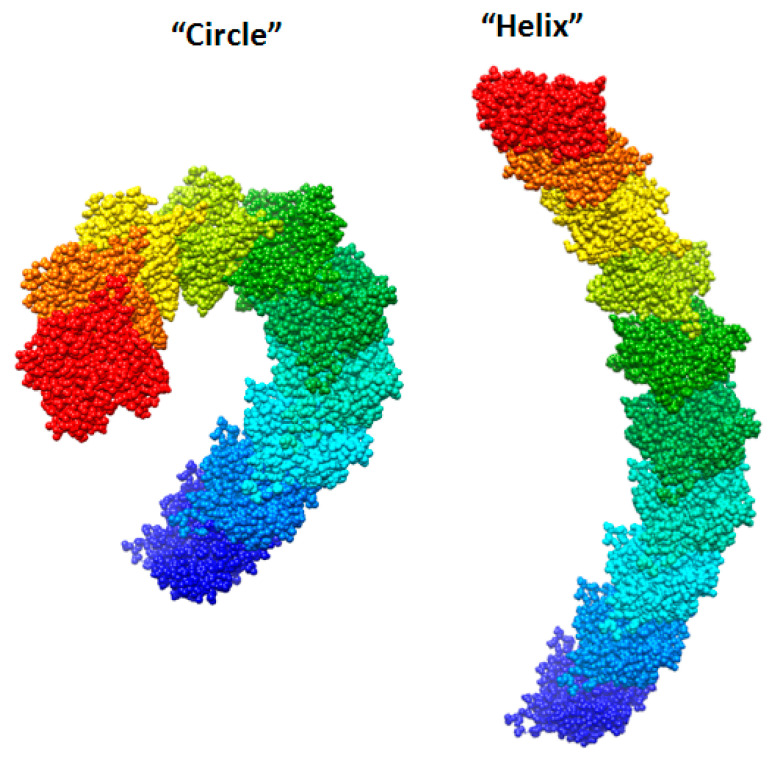
New F-actin models: “circle” and “helix”. Each of these models consists of 10 actin monomers. The PDB structure (2zwh) was taken as a building unit.

We created two models of one filamentous actin in such a way that the regions constituting the F-actin core were as little as possible accessible to the solvent. These patterns were designated as “circle” and “helix” (Figure 7).

**Figure 8:**
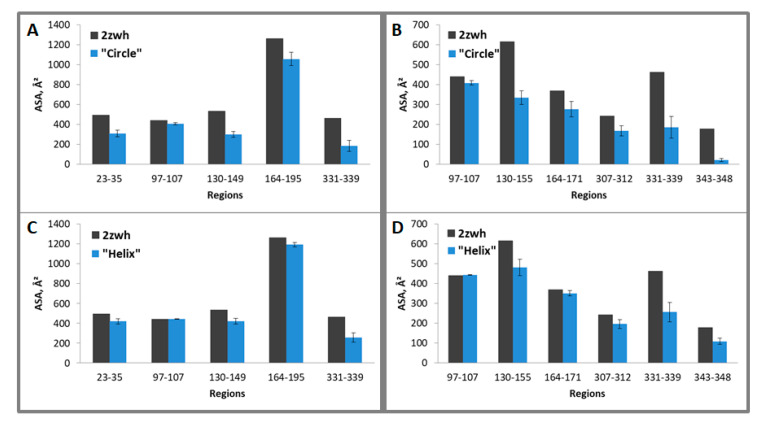
Average accessible surface areas (ASA) of regions 23–35, 97–107, 130–149, 164–195, and 331–339 (for proteinase K) and 97–107, 130–155, 164–171, 307–312, 331–339, 343–348 (for mixture of proteases) for one filamentous “circle” (**A**,**B**) and “helix” (**C**,**D**) models and monomeric actin structure (2zwh). Averaging was carried out for eight monomers. The terminal monomers were not taken into the consideration. These regions were detected by using mass spectrometry after proteolysis of proteinase K (**A**,**C**) and a mixture of proteases (**B**,**D**).

It follows from the calculations that the “circle” model is more consistent with the experimental data for F-actin treatment with proteinase K and a mixture of proteases than the “helix” model (Figure 8). However, the “circle” model contradicts the experimental data of electron microscopy (Figure 2), and the “helix” model is consistent with these data.

### 2.4. X-ray Diffraction Analysis

**Figure 9:**
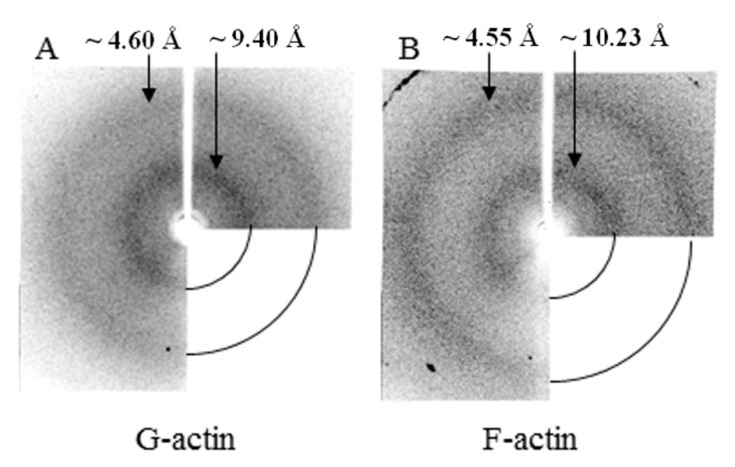
X-ray diffraction pictures of G- (**A**) and F- actin (**B**).

According to the data of X-ray structural analysis, monomeric actin under conditions of 20 mM TrisHCl, pH 7.8, 5 mM ATP, 0.5 mM βME has two diffuse diffractions—equatorial about 4.6 Å and meridian about 9.4 Å (Figure 9A). These diffractions are one of the main characteristics of amyloid fibrils. However, a sufficient amount of data have been accumulated that these diffractions reflect not only the presence of a cross-β structure in fibrils, but also reflect the presence of a β-structure in molecules. X-ray diffraction analysis of F-actin under the conditions of 20 mM TrisHCl, pH 7.8, 5 mM ATP, 0.5 mM βME, 0.1 M KCl also shows two diffuse diffractions that are characteristic of amyloid fibrils—4.55 Å and 10.23 Å (Figure 9B). These diffractions are the same for G- and F-actin (Figure 9).

To date, a lot of data have been accumulated that do not agree with the generally accepted model of F-actin organization. Construction of a new model of actin filament is a necessary step for resolving the accumulated contradictions in the interpretation of a number of results of studies of actin filament. Some controversy may be related to the double-stranded model of the actin filament. Studying the mechanism of actin fibrillation will help clarify the correctness of the proposed new model of F-actin. Our experimental and theoretical data do not agree with the existing model of F-actin, which was proposed in 1963 [40] using electron microscopic images and taking into account the data of X-ray diffraction studies of F-actin [39]. It should be noted that the authors of the X-ray data [39] were unable to draw a conclusion about the double-stranded organization of F-actin. But Hanson and Lowy [40] suggested that F-actin has double-stranded organization with a high probability. This proposed model is insignificant for binding partners, since almost all amino acids of actin involved in interaction with partner proteins are located along the lateral surface of F-actin. However, the existing F-actin model cannot be entirely described by a single structural model, since this model cannot explain the destruction of actin filaments after introduction of disulfide bonds [43] between residues which participated in the contacts between monomers in actin filament according to the double-stranded model of F-actin [41,54,55]. These results indicate a high degree of plasticity and heterogeneity of F-actin. Our understanding of the structural organization of F-actin differs from that accepted today. A ladder-like stacking of G-actin into F-actin (Figure 1, Figure 2 and Figure 3), as we observed for many amyloid-forming peptides and proteins, may explain the strong polymorphism of actin filaments. It should be noted that such an organization of F-actin is presented in the EM images [37,56], but taking into account the double-stranded organization of F-action scientists did not see an evident organization of F-actin.

## 3. Materials and Methods

### 3.1. Isolation of α-Actin from Rabbit Skeletal Muscle and Obtaining G- and F-Forms of Actin

Actin was obtained by the Spudich and Watt 1971 [57] method from acetone powder prepared by the Feuer and Molnar 1948 [58] method. The rabbit muscle fibers were homogenized, followed by myosin extraction with solution (0.3 M KCl, 0.15 M K-phosphate buffer, pH 6.5). Then, the resulting homogenate was treated with 50 mM NaHCO_3_ for 10 min at 4 °C, followed by centrifugation for 30 min at 5000× *g*. The precipitate was suspended at 4 °C in a solution containing 1 mM EDTA, pH 7.0 at 4 °C for 10 min, followed by centrifugation at 5000× *g* for 10 min and washing with distilled water for 5 min at 4 °C. The procedure was repeated twice. Next, acetone powder was prepared from the precipitate by treating the precipitate with pre-cooled acetone at room temperature 5 times for 10 min, followed by filtration through a fabric filter. The resulting residue was dried in air at room temperature for a day, after which it was sieved to separate the actin powder from the stroma and stored at –20 °С.

From the obtained acetone powder, actin was extracted in Spudich and Watt buffer containing 2 mM tris-HCl, 0.2 mM Na_2_-АТP, 0.5 mM βME (β-mercaptoethanol), 0.2 мМ CaCl_2_, pH 8.0. Extraction was carried out on the base of ratio of 20 mL of buffer per 1 g of powder for 25 min on ice with continuous stirring, followed by centrifugation for 5 min at 7000× *g*. Then, the extraction procedure was repeated with a half volume of the buffer, the extracts were combined and clarified by centrifugation for 30 min at 12,000× *g*.

Then actin was polymerized by adding a solution of 3 M KCl to a concentration of 0.05 M and a solution of 1 M MgCl_2_ to a concentration of 2 mM and left during 2 h at 4 °C. Next, a solution of 3 M KCl was added to the polymerized actin to a concentration of 0.6 M and stirred for 1 h at 4°C, followed by centrifugation for 2 h at 100,000× *g*. The precipitate was suspended in a small amount of the original extraction buffer and dialyzed against it for 2 days to depolymerize the actin. The depolymerized actin was clarified by centrifugation for 2 h at 100,000× *g*. The actin concentration was determined by spectrophotometry method using E_280_^1mg/mL^ = 1.09 [59]. SDS-PAGE of purified actin is presented in Figure S2.

### 3.2. Mass Spectrometry Analysis

For actin polymerization, a KCl solution was added to the sample with a concentration of 3 mg/mL to a final concentration of 0.1 M and incubated at 37 °C for a day. Then, the sample containing the F-actin was centrifuged for 20 min at 10,000× *g* in an Eppendorf 5418R centrifuge (Eppendorf, Hamburg, Germany). The pellet was washed twice with 100 mM NH_4_HCO_3_ (pH 7.5). Thus, precipitated fibrils were dissolved in 100 mM NH_4_HCO_3_ (pH 7.5) to a concentration of 1 mg/mL. Then, the mixture was supplemented with CaCl_2_ solution to a concentration of 5 mM to ensure efficient functioning of proteinase K. Both proteinase K and protein mixture (trypsin, chymotrypsin and proteinase K) were used for F-actin proteolysis. Final protein/protease ratio was 25/1 (in the case of protease mixture the ratio was kept for all proteases). After incubation with the protease (mixture of proteases) for 8 h at 37 °C in a Thermomixer comfort (Eppendorf, Hamburg, Germany) at a stirring speed of 450 rpm, the solution was centrifuged at 10,000× *g* during 20 min. The precipitate was isolated and then dissolved with 0.1% trifluoroacetic acid. The samples were then dried using an Eppendorf 5301 vacuum concentrator (Eppendorf, Hamburg, Germany). Proteolysis of the monomeric form of actin was performed similarly but without pelleting and washing.

Mass spectrometry analysis of fractions was performed on a high-resolution mass spectrometer OrbiTrap Elite (Thermo Scientific, Dreieich, Germany). Fragmentation of ions was carried out by the methods of collision activated dissociation (CAD) and electron transfer dissociation (ETD). The mass of ions and ion fragments was recorded with a resolution of 240,000 and 60,000, respectively. The obtained fragmentation spectra were processed using the PEAKS Studio 7.5 software (Bioinformatics Solution Inc., Waterloo, ON N2L 6J2, Canada). Peptides for which the ion current signal intensity was greater than 10^5^ were regarded significant.

### 3.3. Electron Microscopy

EM analysis of G-actin was carried out with a freshly obtained preparation. The sample was obtained according to the method described above. For EM analysis, immediately after isolation of G-actin in buffer 20 mM TrisHCl, pH 7.8, 5 mM ATP, 0.5 mM βME, it was adjusted to a concentration of C = 0.2 mg/mL. To convert G-actin into F-actin, up to 0.1 M KCL was added to the preparation of G-actin and incubated at room temperature for about 20 h (according to EM analysis, after incubation for 5-10 h, not all monomeric actin is converted into filamentous form (Figure S3)).

The concentration of F-actin before EM analysis was adjusted with a buffer with 0.1 M KCl to 0.2 mg/mL. Both preparations were prepared for EM analysis by negative contrast. A copper grid 400 mesh (Electron Microscopy Sciences, Hatfield, PA, USA) coated with a formvar film (0.2% formvar solution in chloroform) was mounted on a sample drop (10 μL). After 5 min absorption, the grid with the preparation was transferred onto a drop (50 μL) of 1% aqueous (weight/volume) solution of uranyl acetate and contrasted for 1.5–2.0 min. The excess staining agent was removed with filter paper. The preparations were analyzed using a JEM-1200 EX transmission electron microscope (Jeol, Tokyo, Japan) at the accelerating voltage of 80 kV. Images were recorded on the Kodak electron image film (SO-163) (Kodak Electron Image Film, New York, NY, USA) at nominal magnification of 40,000–60,000.

### 3.4. Databases and Programs

The list of filamentous actin protein structures available in the Protein Data Bank [60] was taken from the UniProtKB database [61], record number P68135, gene ACTA1, wild rabbit species (*Oryctolagus cuniculus*). The pairwise spatial alignment of 155 actin structures showed that the root mean squire deviations (RMSDs) calculated from the Cα atoms for these pairs do not exceed 3 Å. It follows that the structures of the rabbit actin proteins are very similar.

The calculation of the accessible surface area (ASA) for each amino acid residue in the actin structures and RMSD between structures were performed using the YASARA program [62].

### 3.5. X-ray Diffraction Analysis

Actin preparations for X-ray analysis were prepared according to the procedure described in Serpell et al., 1999. Monomeric actin preparations with concentration C = 0.5 mg/mL under conditions of 20 mM TrisHCl, pH 7.8, 5 mM ATP, 0.5 mM βME. Polymer actin under conditions of 20 mM TrisHCl, pH 7.8, 5 mM ATP, 0.5 mM βME, 0.1 M KCl was either concentrated to C = 5 mg/mL using a vacuum concentrator (Eppendorf 5301 vacuum concentrator, Hamburg, Germany) at room temperature, or actin solutions with initial concentrations of 3.0–4.0 mg/mL were used. Drops (6–8 μL) of the preparations were placed with a gap (about 1.0–1.5 mm) between glass rods (rod diameter 1–1.5 mm), the ends of which were coated with wax and dried for several hours in Petri dishes at room temperature. X-ray diffraction analysis was carried out on an X8 Proteum System X-ray diffraction complex (Bruker AXS, Karlsruhe, Germany) using Cu Kα-radiation (λ = 1.54 Å).

## 4. Conclusions

Studying the mechanism of amyloid fibril formation by the example of a number of peptides, we are approaching the understanding that the formation of fibrils follows the pathway: monomer → oligomer → fibril. Wherein, the oligomer is the main building block for the formation of fibrils. The building blocks in the form of like-ring oligomers interact with each other ring to ring, lining up in the long polymer formations [63]. The actin molecule itself can be a ready-made building block, since its three-dimensional parameters (width, height) correspond to those of F-actin.

We demonstrated that the regions inaccessible for the action of proteinase K and a mixture of proteases (trypsin, chymotrypsin, and proteinase K) are completely open (except for one region 164–195 for proteinase K and 164–171 for a mixture of proteases) in existing models of filamentous actin using the data of proteolysis and mass spectrometry analysis.

The filamentous actin presented in the EM images has no double-stranded organization, which is the generally accepted concept of filamentous actin. We observed ladder-like stacking of G-actin into F-actin in the EM images. Moreover, G- and F-actin have the same X-ray diffractions.

Taking into account the data of proteolysis, mass spectrometry, X-ray diffraction, and electron microscopy a new model of stacking actin monomers in filamentous actin is proposed. According to this model, actin monomers form one filament in such a way that the regions that make up the F-actin core are as little accessible to the solvent as possible.

## Figures and Tables

**Table 1:** Some characteristics of F-actin from the Protein Data Bank.

PDB ID	2w49	1m8q	3g37	3j8k	6bnp
Resolution, Å	35.0	70.0	6.0	12.0	4.6
Method of resolution	EM	EM	Cryo-EM	EM	Cryo-EM
Year	2008	2002	2009	2014	2017
Number of monomers	16	14	12	10	8
Average RMSD between monomers in filament, Å	0	0	0.40	1.29	0.52
Ligand	Tropomyosin	Myosin	None	None	Myosin
Structure	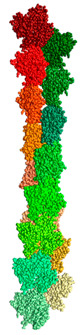	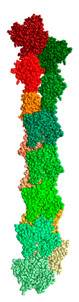	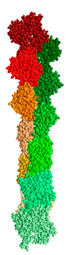	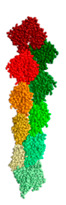	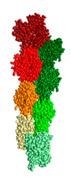

## Supplementary Materials

Supplementary materials can be found at https://www.mdpi.com/1422-0067/21/21/8319/s1. Figure S1. Mass spectrometry analysis of the monomer actin regions protected to the proteolysis treated with mixture of proteases (65–67—black, 102–115—red, 130–134—green, 164–167—magenta, 223–228—brown, 288–292—cyan, 308–312—pink and 331–338—blue) (A) and F-actin regions protected to the proteolysis treated with proteinase K (23–35—orange, 97–107—red, 130–149—green, 164–195—magenta and 331–339—blue) (B) and treated with mixture of proteases: trypsin, chymotrypsin and proteinase K (97–107—red, 130–155—green, 164–171—magenta, 307–312—pink, 331–339—blue, 343–348—gray) (С). Figure S2. SDS-PAGE of purified actin. Figure S3. EM analysis of the kinetics of the transition of G-actin to F-actin: G-actin (A); preparation after 1–5 hours of incubation (B); preparation after 20 hours of incubation in the presence of 0.1 M KCl (C).

## Abbreviations

EM	electron microscopy
RMSD	root mean square deviation
ASA	accessible surface area
βME	β-mercaptoethanol
