# Supplementary material for: New Model for Stacking Monomers in Filamentous Actin from Skeletal Muscles of Oryctolagus cuniculus
Source: Int J Mol Sci. 2020 Nov 6;21(21):8319. doi: 10.3390/ijms21218319 (PMC7664232; doi:10.3390/ijms21218319)
Supplement: Supplementary file 1 [file ijms-21-08319-s001.pdf]

New Model for Stacking Monomers in Filamentous Actin from Skeletal Muscles of *Oryctolagus cuniculus*

Anna V. Glyakina[a,b], Alexey K. Surin[a,c,d], Sergei Yu. Grishin[a], Olga M. Selivanova[a], Mariya Yu. Suvorina[a], Liya G. Bobyleva[e], Ivan M. Vikhlyantsev[e], Oxana V. Galzitskaya[a,e*]

[a] Institute of Protein Research, Russian Academy of Sciences, Pushchino, Moscow Region, Russia; ogalzit@vega.protres.ru

[b] Institute of Mathematical Problems of Biology, Russian Academy of Sciences, Keldysh Institute of Applied Mathematics, Russian Academy of Sciences, Pushchino, Moscow Region 142290, Russia; quark777a@gmail.com

[c] The Branch of the Institute of Bioorganic Chemistry, Russian Academy of Sciences, Pushchino, Moscow Region, Russia; alan@vega.protres.ru

[d] State Research Center for Applied Microbiology and Biotechnology, Obolensk, Russia; alan@vega.protres.ru

[e] Institute of Theoretical and Experimental Biophysics, Russian Academy of Sciences, Pushchino, Moscow Region, Russia; ivanvikhlyantsev@gmail.com

* Correspondence: ogalzit@vega.protres.ru; Tel.: (+7-903-675-0156 (O.V.G.))

Supplementary materials:

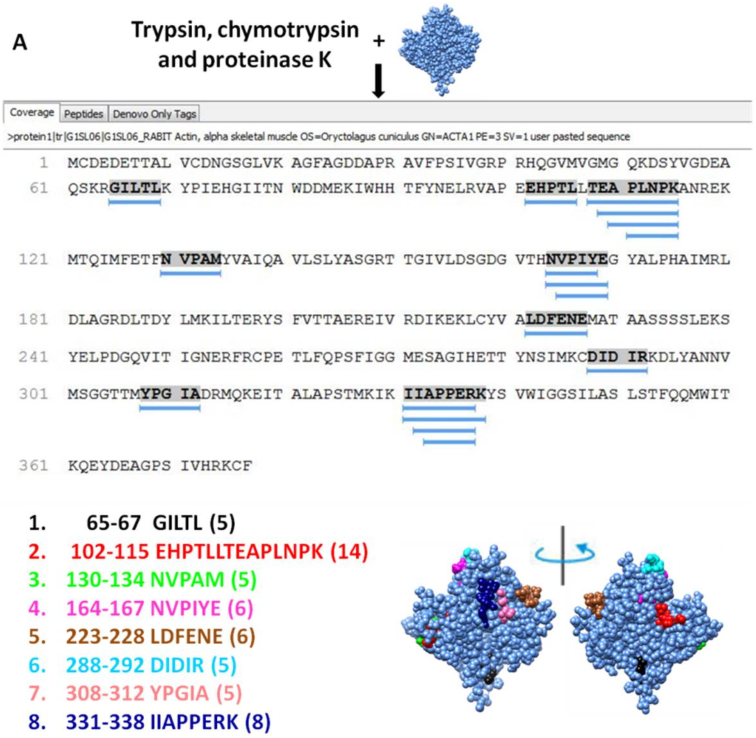

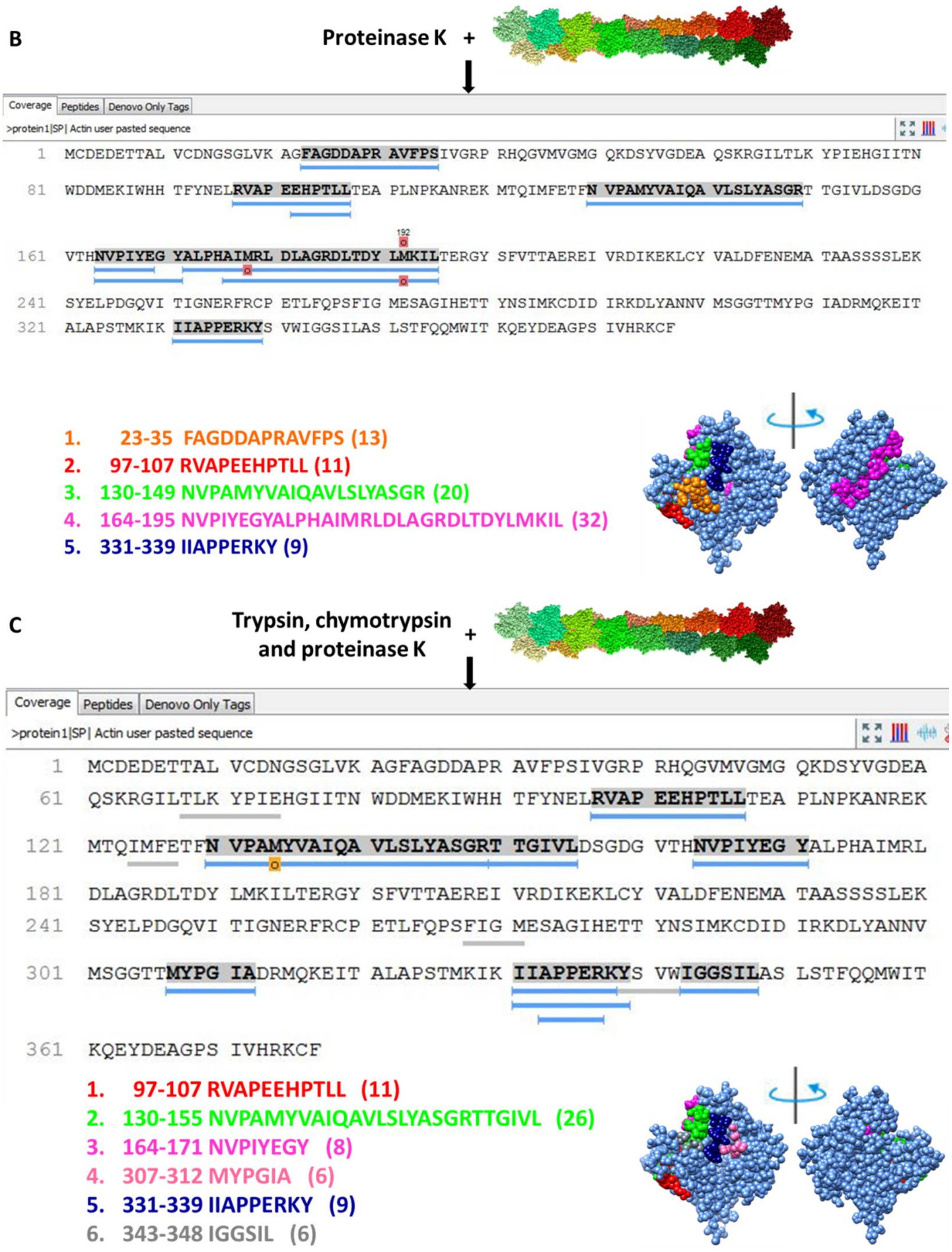

Figure S1. Mass spectrometry analysis of the monomer actin regions protected to the proteolysis treated with mixture of proteases (65-67 – black, 102-115 – red, 130-134 – green, 164-167 – magenta, 223-228 – brown, 288-292 – cyan, 308-312 – pink and 331-338 – blue) (A) and F-actin regions protected to the proteolysis treated with proteinase K (23-35 – orange, 97-107 – red, 130-149 – green, 164-195 – magenta and 331-339 – blue) (B) and treated with mixture of proteases: trypsin, chymotrypsin and proteinase K (97-107 – red, 130-155 – green, 164-171 – magenta, 307-312 – pink, 331-339 – blue, 343-348 – gray) (C).

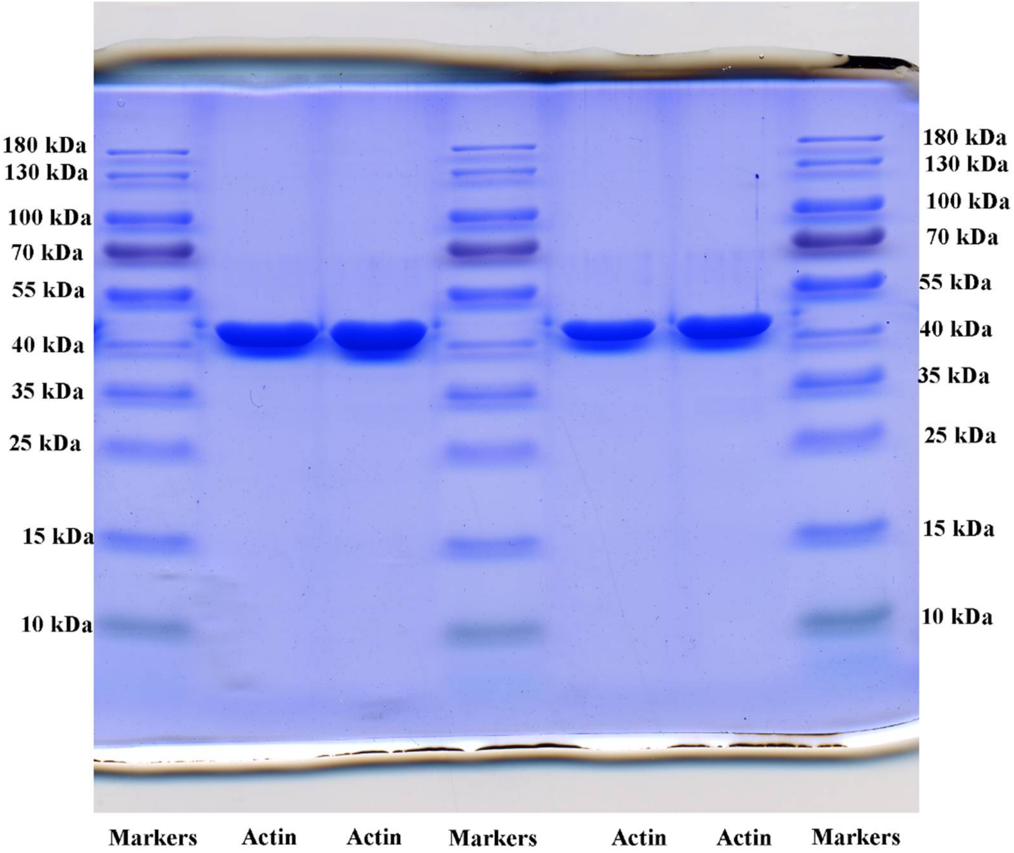

Figure S2. SDS-PAGE of purified actin.

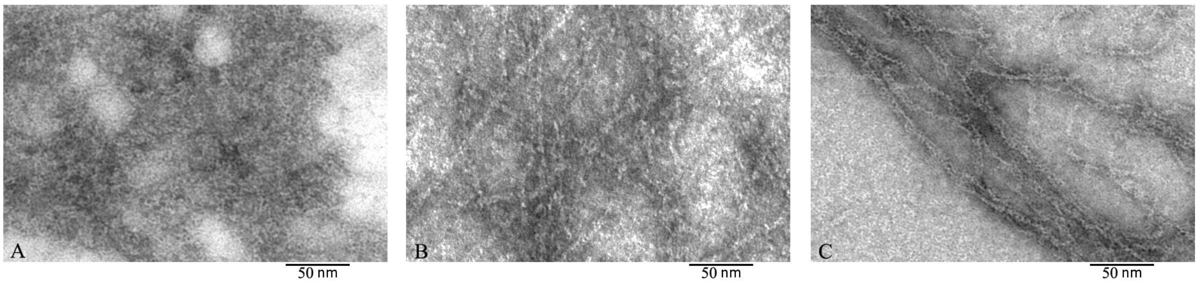

Figure S3. EM analysis of the kinetics of the transition of G-actin to F-actin: G-actin (A); preparation after 1-5 hours of incubation (B); preparation after 20 hours of incubation in the presence of 0.1 M KCl (C).
